# Losing women along the path to safe motherhood: why is there such a gap between women’s use of antenatal care and skilled birth attendance? A mixed methods study in northern Uganda

**DOI:** 10.1186/s12884-015-0695-9

**Published:** 2015-11-04

**Authors:** Erin Anastasi, Matthias Borchert, Oona M. R. Campbell, Egbert Sondorp, Felix Kaducu, Olivia Hill, Dennis Okeng, Vicki Norah Odong, Isabelle L. Lange

**Affiliations:** United Nations Population Fund (UNFPA), 605 Third Avenue, New York, NY 10158 USA; London School of Hygiene & Tropical Medicine, London, UK; Institutes of Tropical Medicine and International Health, Charité-Universitätsmedizin Berlin, Berlin, Germany; Gulu University Faculty of Medicine, Gulu, Uganda; Medicos sin Fronteras (MSF/Doctors Without Borders) - Spain/OCBA, Barcelona, Spain; Apac Hospital, Apac, Uganda

**Keywords:** Maternal health, Antenatal care, Delivery care, Maternal/newborn care, Health services, Quality of care, Uganda

## Abstract

**Background:**

Thousands of women and newborns still die preventable deaths from pregnancy and childbirth-related complications in poor settings. Delivery with a skilled birth attendant is a vital intervention for saving lives. Yet many women, particularly where maternal mortality ratios are highest, do not have a skilled birth attendant at delivery. In Uganda, only 58 % of women deliver in a health facility, despite approximately 95 % of women attending antenatal care (ANC).

This study aimed to (1) identify key factors underlying the gap between high rates of antenatal care attendance and much lower rates of health-facility delivery; (2) examine the association between advice during antenatal care to deliver at a health facility and actual place of delivery; (3) investigate whether antenatal care services in a post-conflict district of Northern Uganda actively link women to skilled birth attendant services; and (4) make recommendations for policy- and program-relevant implementation research to enhance use of skilled birth attendance services.

**Methods:**

This study was carried out in Gulu District in 2009. Quantitative and qualitative methods used included: structured antenatal care client entry and exit interviews [*n* = 139]; semi-structured interviews with women in their homes [n = 36], with health workers [n = 10], and with policymakers [n = 10]; and focus group discussions with women [n = 20], men [n = 20], and traditional birth attendants [n = 20].

**Results:**

Seventy-five percent of antenatal care clients currently pregnant reported they received advice during their last pregnancy to deliver in a health facility, and 58 % of these reported having delivered in a health facility. After adjustment for confounding, women who reported they received advice at antenatal care to deliver at a health facility were significantly more likely (aOR = 2.83 [95 % CI: 1.19–6.75], *p* = 0.02) to report giving birth in a facility. Despite high antenatal care coverage, a number of demand and supply side barriers deter use of skilled birth attendance services. Primary barriers were: fear of being neglected or maltreated by health workers; long distance and other difficulties in access; poverty, and material requirements for delivery; lack of support from husband/partner; health systems deficiencies such as inadequate staffing/training, work environment, and referral systems; and socio-cultural and gender issues such as preferred birthing position and preference for traditional birth attendants.

**Conclusions:**

Initiatives to improve quality of client-provider interaction and respect for women are essential. Financial barriers must be abolished and emergency transport for referrals improved. Simultaneously, supply-side barriers must be addressed, notably ensuring a sufficient number of health workers providing skilled obstetric care in health facilities and creating habitable conditions and enabling environments for them.

## Background

Nearly 300,000 women worldwide die from pregnancy and childbirth-related causes each year [1]. Most of these deaths are avoidable, as solutions for preventing them are well-known [2]. Disparities in accessing preventive interventions are particularly challenging in remote areas with high poverty. Of all health indicators, maternal mortality reveals the greatest disparity between rich and poor countries [3]. Skilled birth attendance (SBA) during labour and delivery has been identified as “the single most important factor in preventing maternal deaths” [4], and is also very unequal between the richest and poorest quintiles within countries.

In Uganda, maternal mortality is estimated at 360 deaths per 100,000 live births [5]. Evidence suggests that inequities in access to health services have grown over the last decade, while key health indicators, including maternal and infant mortality rates, have stalled or worsened among the poor [6, 7]. The percentage of births in Uganda with SBA is 88 % in the wealthiest quintile, but only 44 % in the poorest [6, 8]. Regional and urban-rural inequities persist. Percentages of health facility births are estimated at 90 % in urban areas but 53 % in rural settings. Met need for emergency obstetric care, captured via the numbers of EMOC facilities per 100,000 population is estimated at 24 % nationally, but 14 % in the North [6, 9].

Global data show clear evidence that antenatal care (ANC) attendance is associated with higher SBA utilisation [10, 11]. There appears to be a “dose–response” relationship between ANC and SBA, with women receiving ANC “early and often” being most likely to also receive SBA [12–17]. While association does not imply causation, data indicate that in low and middle-income countries, SBA is six times more likely among women who attended ANC at least once during pregnancy than among women who did not attend at all [11].

However, research data also demonstrate a gap between a much higher level of ANC attendance than of SBA utilization [10, 11]. The gap has been confirmed in Africa [18–24] where it appears most persistent and pronounced [10, 11], and elsewhere [25–28]. Stekelenburg et al. [29] concluded, “The large difference between the reasonably high ANC attendance and the lower supervised delivery percentage is still not fully understood”.

At the Médecins Sans Frontières (MSF)-supported health centre in Lalogi (Gulu district), service statistics illustrate the existence of the “ANC-SBA gap”. While the annual number of first ANC visits and deliveries has held relatively constant over the years, the disparity between the annual number of new ANC visits and the annual number of health facility deliveries is approximately 3:1 or even 4:1 (Table 1). Women were apparently able to access health services despite the conflict, but far greater proportions used ANC than SBA.

**Table 1:** Number of annual ANC and delivery visits in Lalogi, Uganda

Summary - annual service statistics - Lalogi, Uganda - MSF/Spain/Uganda MoH			
Indicator (annual total)	2006	2007	2008*
total # first ANC visits/year	1150	2250	2711
total # ANC visits (first + follow up)/ year	1790	4264	5824
total # deliveries/year	435	491	577
ratio first ANC visits/deliveries	2.6:1	4.6:1	4.7:1

*2008 data are extrapolated from the period Jan. - Sept

Our study sought to examine the reasons for the gap between women’s use of ANC and SBA services (typically only available in health facilities) in Northern Uganda, where approximately 95 % of pregnant women use ANC but only 54 % deliver at a health facility [5]. Specifically, the study focused on identifying reasons why this gap existed even in the presence of a fully-staffed and equipped MSF-supported health centre, with free, 24-hour 7-day-a-week SBA services. The study also aimed to assess and take into account women’s and community perceptions, experiences, and needs [30].

This study aimed to (1) identify key factors underlying the gap between high rates of ANC attendance and much lower rates of health facility delivery; (2) examine the association between health workers’ advice at ANC to deliver at a health facility and the actual place of last delivery; (3) investigate whether ANC services attempt to actively link women to SBA services in the conflict-affected Gulu District of Northern Uganda; and (4) make recommendations for policy- and program-relevant implementation research to enhance use of skilled birth attendance services.

## Methods

### Study setting

Civilians in Northern Uganda were living under war conditions for more than 20 years, and the area has only in recent years emerged from conflict. Residents of Gulu District faced massive displacements since 1996 resulting from the Lord’s Resistance Army (LRA) insurgency, which targeted civilians, resulting in tremendous and prolonged suffering. In mid-2006, the Government of Uganda and the LRA signed a Cessation of Hostilities Agreement. Since November 2006, Gulu District Authorities have encouraged Internally Displaced Persons’ (IDP) to return to their villages.

This study targeted a public-sector Ministry of Health (MoH) facility (“Lalogi”) in Northern Uganda’s Gulu District, supported by MSF. The facility was located inside an IDP camp to eliminate distance and insecurity as barriers to service utilization.

### Study design

The study employed a quantitative and qualitative approach. Quantitative methods were used to assess whether advice during ANC consultation was associated with increased facility delivery. Qualitative methods were used to understand the dynamics of decision-making by clients, communities, and providers regarding where women give birth.

The following data collection techniques were used:

1. Structured entry and exit interviews with ANC clients at Lalogi health centre;
2. Home-based semi-structured interviews with women who attended ANC and gave birth (either at home or in a health facility) within the past 2 years;
3. Semi-structured interviews with local facility-based maternity care providers;
4. Semi-structured interviews with Ugandan policymakers or key stakeholders at sub-district, district, and national levels;
5. Focus group discussions (FGD) with women, men, and traditional birth attendants in catchment communities.

### Conceptual framework

**Fig. 1:**
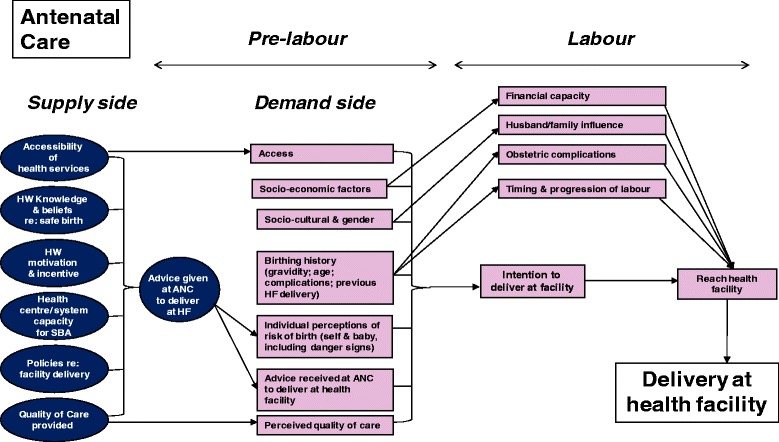
Please see separate file (Fig. 1: ANC-SBA link/gap model: Factors influencing the link or gap between women’s use of ANC and SBA services) uploaded through the online submission system

The study investigated both demand- and supply-side barriers to utilization of SBA among women who have already used ANC services (see Fig. 1: ANC-SBA link/gap model: factors influencing the link or gap between women’s use of ANC and SBA services, in separately uploaded file). The guiding framework drew on existing studies [46, 49] in describing barriers women face in accessing quality preventive or routine intrapartum care and emergency obstetric care. However, the conceptual framework for this study attempted to integrate roles and perspectives from both the demand-side (women) and the supply-side (ANC providers). Thus, the framework includes several health systems factors not included in the above-referenced studies – namely, health worker knowledge, beliefs, motivation, and incentives, and whether they advise ANC clients to deliver in facilities; health system capacity for SBA; policies regarding SBA – as well as some demand-side factors such as woman’s birthing/obstetric history; whether she was advised at ANC to delivery in a health facility; the timing and progression of labour; and her perception of the quality of care available at health facilities.

### Study population, selection, and recruitment of participants

For the quantitative client entry and exit interviews, data were obtained from women visiting the ANC clinic for the first time during their current pregnancy. All such women were eligible, and the research team attempted to interview all eligible women each day data collection was ongoing.

A sample size of 130 women was selected to detect an odds ratio of 4 for the association between having received ANC advice and actually having delivered at a health facility at last birth, assuming 15 % received advice and about 20 % of women who did not receive advice would deliver in a health facility. A total of 139 women were recruited for entry-exit interviews at Lalogi health centre.

For the qualitative interviews with ANC clients who had delivered within the previous two years, sampling was purposive, to assess the scope of views, which were expected to vary with parity and place of delivery at last birth. The final sample size (*N* = 36) was determined using a saturation approach. The sampling grid (Table 2) defined profiles of women of reproductive age (15–49 years) selected for interview [31]. For the qualitative interviews, women were purposively selected. Starting at the gate of Lalogi Health Centre IV, researchers randomly selected a direction and walked in that direction in Lalogi camp, within a maximum walking distance of 20 min. While walking, researchers asked around at households, markets, and community gathering sites for women who fit the eligibility criteria. Subsequently, the “snowball” technique was used to identify additional women who met the criteria. Selection of women involved stratification based on whether the woman’s last delivery was at a health facility or at home, and parity. Selection of interviewees also involved restricting for distance, i.e., selecting only women who lived within reasonable access to the health facility and for whom distance was unlikely to be the primary reason for non-use of the facility. These qualitative interviews were conducted with women in their own homes. For example, after locating a woman in the market who met the eligibility criteria, researchers arranged a time to go to her home for an interview, typically the following day. While there is always a possibility of selection bias, the above- described measures aimed to minimize this possibility, and researchers are not aware of any women who refused to participate in interviews or FGDs.

**Table 2:** Sampling grid of home-based interviews with women

	Delivered at health facility (most recent birth)	Did not deliver at health facility (most recent birth)	Total
Primipara (1)	5	6	11
Multipara (2+)	14	11	25
Total	19	17	36

Ten semi-structured interviews were conducted with health workers providing maternity care; ten interviews with Ugandan policymakers at sub-district, district, and national levels, selected based upon local co-investigator recommendations and the snowball technique; and six focus group discussions (two with women, two with men, and two with traditional birth attendants) were carried out, comprising 60 participants in total. Recruitment criteria for FGD participants included the following: women in the community surrounding Lalogi who had given birth at least once within the past 2 years; men in the Lalogi community whose wives/partners had given birth in the past 2 years; and TBAs working in partnership with Lalogi Health Centre.

### Data collection

Eligible women had a brief interview before entering, and after exiting, the ANC clinic. Content and perceived quality of ANC counselling were addressed in the exit interviews. Interviews were conducted every day during nearly two months to capture a diverse sample of clients receiving ANC services from different providers.

Interview schedules for the semi-structured interviews and topic guides for FGDs were adapted from existing tools [32–36]. The tools were translated and back-translated as needed, then piloted and adapted accordingly. Interviews and FGDs were conducted in local language (Acholi and/or Langi) by the trained, local research assistants. All sessions were recorded, transcribed, translated, and back translated to ensure accuracy and quality.

### Data management and analysis

We examined the association of several supply- and demand-side factors with the primary outcome of giving birth in a health facility. Quantitative data were double-entered with EpiData (EpiData Association, Odense, Denmark), checked for consistency and validity, and analysed with Stata 10 (StataCorp, College Station, Texas, USA).

Following univariable and bivariable analyses, multivariable logistic regression analyses were performed to assess: a) factors associated with women receiving advice at ANC to deliver at a health facility; b) factors associated with previous health facility delivery; and c) the association between ANC advice and place of delivery.

In bivariable analysis, crude odds ratios together with 95 % confidence intervals were calculated, and proportions were compared using chi-square test. Stratified analysis was conducted to explore confounding and interaction. In multivariable analysis we used manual forward selection, comparing odds ratios to identify confounders, and performing likelihood ratio tests to identify secondary exposures. In addition to the primary exposure (ANC advice to deliver in a health facility), woman’s education and cost of reaching health services were identified a priori from the literature as potentially important exposures or confounders, and were tentatively included in the multivariable logistic regression model. Gravidity and ethnic group, which had been identified as exposures or confounders in bivariable or stratified analysis, were tentatively included in the model as well. Variables, which had a high proportion of missing values, or which in multivariable analysis turned out to be neither confounder nor secondary exposure, were ultimately removed from the model.

For qualitative data, topic guides for semi-structured interviews and focus groups were developed based on themes essential to answering the research questions. A coding scheme was developed, expanded, and modified as the study progressed. Transcripts, observers’ notes, and facilitators’ reports were synthesized and analysed, using a framework approach. Analysis questionnaires and matrices were developed for each group of respondents. We tracked the frequency of recurring themes and compared themes across differing respondent groups to determine patterns, similarities, and differences. Based on frequency, content, and strength of respondents’ statements, factors were assigned relative weight in terms of their impact on ANC clients’ use of SBA services.

### Ethics

Ethics approval for the study was obtained from the London School of Hygiene & Tropical Medicine, Gulu University, the Uganda National Council for Science and Technology, and MSF. Written informed consent was requested and obtained from all participants, who were assured of confidentiality of any information given.

## Results

### Characteristics of the quantitative study sample

Socio-demographic, reproductive and obstetric characteristics of the quantitative sample are presented in Table 3.

**Table 3:** Socio-demographic characteristics of women visiting ANC at Lalogi hospital for the first time in the current pregnancy, 2009

	Frequency	Percent
Age (*n* = 136)*		
15–19	19	14.0
20–24	40	29.4
25–29	38	27.9
30–34	26	19.1
35–45	13	9.6
Education (*n* = 136)*		
Woman’s education		
No formal education	26	19.1
Incomplete primary	90	66.2
Completed primary	17	12.2
Some secondary (or above)	03	2.2
Husband’s education		
No formal education	10	7.4
Incomplete primary	50	36.8
Completed primary	34	25.0
Some secondary (or above)	42	30.9
Ethnic group (*n* = 136)*		
Acholi	69	50.7
Langi	67	49.3
Distance to nearest health facility offering delivery services (*n* = 113)*		
<2 km	14	12.4
2–<3 km	21	18.6
3–5 km	39	34.5
>5 km	39	34.5
Time to reach nearest health facility offering delivery services (*n* = 119)*		
<2 h	42	35.3
2–3 h	65	54.6
>3 h	12	10.1
Time to reach Lalogi health facility (*n* = 132)*		
<= 1 h	31	22.3
>1–3 h	82	59.0
>3 h	19	13.7
Cost of reaching nearest delivery care site (*n* = 100)**		
0–1,000	22	22.0
1,001–2000	23	23.0
2,001–3,000	23	23.0
3,001–5,000	21	21.0
>5,000–15,000	11	11.0

* *n* < 139 because of missing values

** 1000 UGX = $0.5 USD

Three-quarters (76.4 %) of women were between 20 and 34 years old. One-fifth had had no formal education, two-thirds had attended but not completed primary education, and only 2.2 % had gone beyond primary. Women’s husbands had higher levels of education, with far greater proportions completing primary level or above (56 % husbands vs. 15 % women, *p* < 0.001) or having at least secondary level education (31 % husbands vs. 2 % women, *p* < 0.001). Respondents were equally divided between the two predominant ethnic groups, Acholi and Langi.

Twelve percent of women lived within 2 km of a health facility providing delivery care, while one-third lived more than 5 km away. One-third reported being able to reach the nearest health facility providing delivery care in less than 2 h, and 90 % within 3 h; 22 % were able to reach Lalogi health centre within 1 h. The cost of reaching delivery care varied from 500 to 15,000 Ugandan shillings (UGX), or approximately $0.25 to $7.50 USD, though only 11 % reported a cost of above 5,000 UGX ($2.50 USD).

### Birthing history and advice received at antenatal care regarding SBA

Selected reproductive and obstetric characteristics are presented in Table 4. Three-quarters of women confirmed that they were counselled by a health worker to deliver in a health facility during their most recent completed pregnancy; 58 % reported actually delivering in a facility. Approximately 11 % reported ever having had a C-section, stillbirth, or fits.

**Table 4:** Reproductive health history of women visiting ANC at Lalogi hospital for the first time in the current pregnancy, 2009

	Frequency	Percent
Gravidity (*n* = 138)*		
1	14	10.1
2	28	20.1
3	15	10.8
4	19	13.7
5	24	17.3
6–7	19	13.7
8–10	19	13.7
In women of gravidity ≥ 2		
Advice received at ANC during last pregnancy to deliver at HF (*n* = 121)**	89	73.6
Place of last delivery: health facility (*n* = 122)**	71	58.2
Ever had C-section/stillbirth/fits (*n* = 123)**	14	11.4

* *n* < 139 because of missing values

** *n* < 124 because of missing values

### Decision-making related to current pregnancy and childbirth: socio-cultural, gender, and husband/family influence

Two-thirds of the women had their first ANC visit in the 2^nd^ trimester. One-quarter of women indicated they would decide for themselves where to deliver, while 32 % said their husband or partner would decide, 30 % said the decision would be taken jointly between themselves and their partner, and 11 % indicated others would decide – including mother, mother-in-law, other family member, or traditional birth attendant (Table 5). Almost all women (98 %) stated their intention to deliver this pregnancy at a health facility.

**Table 5:** Characteristics and decision-making related to current pregnancy (Lalogi, *n* = 136)*

	Frequency	Percent
Length of pregnancy		
0–3 months	13	9.6
4	32	23.5
5	25	18.4
6	37	27.2
7+	29	21.3
Who decides where woman will give birth?		
Self	36	26.5
Husband or partner	43	31.6
Self & husband/partner together	41	30.2
Other	16	11.5
Intended place of delivery for current pregnancy		
Health facility	133	97.8
With TBA (home)	3	2.2

* *n* < 139 because of missing values

### Factors associated with choice of birth setting: advice at ANC, socio-cultural and birthing history factors

The quantitative arm of this study examined whether a number of supply- and demand-side (as laid out in the conceptual framework) factors were associated with giving birth in a health facility [Table 6]. Of those assessed, only the primary exposure – advice to deliver in a health facility – and ethnic group and gravidity were associated in the crude analysis.

**Table 6:** Bivariable analysis of the association between various factors and giving birth in health facility for the last delivery (Lalogi, *n* = 121)**

Factor	Delivery in health facility	*p*-value*	Crude Odds Ratio
			[95 % Confidence Interval]
Woman’s highest level of education	*n* = 71	*p* = 0.92	
No formal education	14/25 (56.0 %)		1
Incomplete primary	47/81 (58.0)		1.09 [.44–2.70]
Completed primary (or above)	10/16 (62.5)		1.31 [.36–4.82]
Husband’s highest level of education	*n* = 71	*p* = 0.46	
No formal education	6/9 (66.7)		2.09 [0.45–9.67]
Incomplete primary	22/45 (48.9)		1
Completed primary	20/32 (62.5)		1.74 [0.68–4.46]
Some secondary (or above)	23/36 (63.9)		1.85 [0.74–4.61]
Age	*n* = 71	*p* = 0.34	
15–19	7/9 (77.8)		1.75 [0.31–10.02]
20–24	24/36 (66.7)		1
25–29	20/38 (52.6)		0.56 [0.21–1.45]
30–34	12/26 (46.2)		0.43 [0.15–1.25]
35–45	8/13 (61.5)		0.80 [0.21–3.03]
*Ethnic group*	*n* = 71	*p* = 0.01	
Acholi	44/64 (68.8)		2.53 [1.18–5.41]
Langi	27/58 (46.6)		1
Religion	*n* = 69	*p* = 0.54	
Catholic	51/84 (60.7)		1
Protestant	18/33 (54.6)		.78 [.34–1.76]
Gravidity	*n* = 124	*p* = 0.02	
2	20/28 (71.4)		1
3–4	24/34 (70.6)		0.96 [0.32–2.92]
5+	27/59 (45.8)		0.34 [0.12–0.92]
Who decides where woman gives birth	*n* = 71	*p* = 0.95	
Self	21/34 (61.8)		1
Husband/partner	23/41 (56.1)		.79 [.31–2.01]
Self & husband/ partner together	20/34 (58.8)		.88 [.33–2.36]
Other	7/13 (53.9)		.72 [.20–2.67]
Woman needs permission to attend health centre		*p* = 0.16	1.70 [.81–3.6]
Yes	46/73 (63.0)		
No	24/48 (50.0)		
Perceived ease of reaching nearest health facility offering delivery care	*n* = 66	*p* = 0.27	
Very difficult	13/21 (61.9)		1
Difficult	36/54 (66.7)		1.23 [.43–3.53]
Easy	13/28 (46.4)		.53 [.16–1.73]
Very easy	4/5 (80.0)		2.46 [.22–28.12]
Distance to nearest health facility offering delivery care	*n* = 59	*p* = 0.44	
Less than 2 km	8/12 (66.7)		2.22 [0.47–10.50]
2–<3 km	9/19 (47.4)		1
3–5 km	23/34 (67.7)		2.32 [0.71–7.61]
>5 km	19/35 (54.3)		1.32 [0.43–4.10]
Time it takes to reach this health facility (Lalogi)	*n* = 71	*p* = 0.14	
Under 1.5 h	16/29 (55.2)		1
1.5–3 h	47/73 (64.4)		1.47 [0.61–3.55]
>3 h	8/18 (44.4)		0.65 [0.20–2.16]
Time to reach nearest HF offering delivery services	*n* = 63	*p* = 0.45	
Under 1.5 h	18/35 (51.4)		1
1.5–3 h	38/59 (64.4)		1.71 [.72–4.05]
>3 h	7/11 (63.6)		1.65 [.40–6.83]
Average fee (UGX) from home to nearest HF offering delivery services	*n* = 59	*p* = 0.79	
0–1,000	11/18 (61.1)		1
1,001–2000	16/22 (72.7)		1.70 [.44–6.62]
2,001–3,000	16/23 (69.6)		1.46 [.39–5.45]
3,001–5,000	10/18 (55.6)		.80 [.21–3.06]
>5,000–15,000	6/10 (60.0)		.96 [.19–4.78]
Woman’s knowledge of danger or warning signs during labour & delivery	*n* = 71	*p* = 0.63	
0/none	23/38 (60.5)		1
1 danger sign	26/47 (55.3)		0.81 [0.34–1.94]
2 danger signs	6/13 (46.2)		0.56 [0.15–2.04]
3 or more danger signs	16/24 (66.7)		1.30 [0.44–3.84]
Previous complications – ever experienced C-section, stillbirth, or fits	(*n* = 71)	*p* = 0.63	
Yes	9/14 (64.3)		1.34 [.42–4.28]
No	62/108 (57.4)		1
Whether woman received advice at ANC during last pregnancy to deliver at health facility	*n* = 71	*p* = .005	
Yes	59/89 (66.3)		3.28 [1.37–7.85]
No	12/32 (37.5)		1

* Derived from chi-square test

** While total number of women interviewed was 139, 18 respondents were missing data on advice, health facility delivery, or both, bringing the denominator to 121

In multivariable analysis, ultimately two variables – gravidity and ethnic group – were maintained in the model in addition to the primary exposure ANC delivery advice. Adjusted ORs are presented in Table 7.

**Table 7:** Multivariable analysis – unadjusted and adjusted odds ratios – factors associated with delivering in health facility at last birth (Lalogi)

Factor	Unadjusted OR [95 % CI]	Adjusted OR [95 % CI]
		Model including ANC delivery advice, ethnic group & gravidity (*n* = 120)
Whether woman received advice at ANC during last pregnancy to deliver at health facility	3.28 [1.37–7.85]	2.81 [1.12–7.01]
Ethnic group *p* = 0.88*		
Langi	1	1
Acholi	2.53 [1.18–5.41]	1.94 [0.86–4.37]
Gravidity *p* = 0.03**		
2	1	1
3–4	0.96 [0.32–2.92]	0.91 [0.28–3.00]
5+	0.34 [0.12–0.92]	0.33 [0.12–0.94]

xi: logistic wherelastbirth advicelast tribe2 i.gravidity2

*p-value from likelihood ratio test, adding ethnic group to the model with advice only

**p-value from likelihood ratio test, adding gravidity to the model with advice and ethnic group only

Gravidity was associated with health facility delivery (likelihood ratio test, *p* = 0.03) and therefore qualified as a secondary exposure, but did not confound the association between ANC delivery advice during last pregnancy and actual delivery at a health facility. Woman’s education was not associated with delivery at a health facility (*p* = 0.84), nor was cost of reaching the nearest health facility offering delivery services (*p* = 0.43). The association between ANC delivery advice and place of last delivery became stronger after adjusting for cost (crude OR = 3.28 vs. adjusted OR = 6.58), but cost data were missing for 20 % of the observations, so that estimates of adjusted odds ratios became imprecise (data not shown); cost was therefore dropped from the model.

Even after controlling for ethnic group and gravidity, there remained significant evidence (*p* = 0.02) of an association between ANC advice to deliver at a health facility and subsequent delivery at a health facility. Women who reported having received this advice during their last pregnancy had nearly three times the odds (OR = 2.81; 95 % CI 1.13–7.01) of reportedly delivering at a health facility, compared to those who reported not having received such advice.

### Voices from the villages: Why is there such a gap between women’s use of ANC and SBA? (Qualitative results)

Based on interviews, we identified the following fundamental reasons underlying the “ANC-SBA gap” in order of importance: poor perceived quality of SBA care, including women’s fear or experience of being mistreated or neglected by health workers, coupled with increased vulnerability during labour and delivery; lack of timely access to health facilities (long distances, lack of transport, limited window of time during labour); poverty, including inability to procure all essential items for health facility delivery (so-called “requirements”); health systems deficiencies, including shortage of competent, motivated and adequately remunerated midwives/skilled birth attendants, as well as lack of essential medicines, supplies, and ambulances; lack of early and adequate birth planning and preparation, together with abrupt or sudden onset of labour and delivery; socio-cultural issues, including lack of perceived need for health facility delivery; and lack of support and active involvement – whether physical, financial, logistic, or emotional – from husbands or male partners. Barriers are discussed below. [Note: quotations are from female FGD and interview participants, unless otherwise noted].

### Quality of Care: Fear, shame, and maltreatment as barriers to health facility delivery

A number of respondents poignantly described their fear or past experience of being hurt, shamed, humiliated, or treated negligently at the hands of health workers. One respondent described women finding themselves *“as a second-hand class of people who can be handled any which way.”* Another responded: *“When you don’t have good, attractive things like clothes, they will just ignore you at the hospital, at times [using] abusive words....When another woman didn’t have a [blanket/clothing] to carry her baby, the nurse started calling her ‘Lacweta’ [pullover], when she was carrying the baby using a pullover. So such words deter women from going to the hospital.”*

Another woman described being “*a laughing stock”* (in the eyes of health workers and peers), a view confirmed by a male focus group participant: *“If I go with my wife when she is not looking very well and without other requirements that health workers need, sometimes the language used is not good…. [health workers may] abuse or look down on you. Sometimes [there are a lot of] people around, but they [use] such bad words, and people laugh at you while you die of shame.”*

Respondents expressed fear of being neglected and invisible if they go to the hospital for delivery. As one woman put it: *“[Women] fear, yet it’s not fear. At times, she could have observed something bad in the hospital. That can stop a woman from going back to deliver. Many think, ‘This hospital, even if I go, nobody will help me. I’d better deliver at home.’ Then she just delivers [at home].”* Another woman spoke of the trauma experienced by many women who found they were left alone or abandoned in their hour of need: *“Women do not like the welcome of the health workers when they go to the hospital…. They will just pass by you as if they have not seen you. [They] are blind to see people….”* And another echoed*: “Even if you are too sick and very weak… the [health workers] should help you. But you find [they are] gone. [They] come back at the time when you are about to die. That is why deaths are so many.”*

### Access Barriers, timing and progression of labour, and a critical window of time

Although access can pose a problem for ANC, the problem becomes more critical during labour and delivery. The delivery site may be farther from home than sites capable of providing ANC; the woman is often weak and in pain and cannot walk far, especially alone; and she is racing against time.

Lack of available and affordable transport, including a functional referral system, is a critical concern. As one female health worker vehemently explained, lack of access can present a life-or-death barrier in emergency referrals:

*"You need to give money summing 30,000 UGX [~$15 USD] onward for fuel [for the ambulance]. [If] you fail to produce it, you [stay behind] and die. You are referred and that is the problem [of] the mother who cannot afford getting such amount.... It's not catered for by the policy - because if it was so, the distributed ambulances, they know that they are going to [need] fuel, and now they are saying there's no fuel”.*

From a policymaker’s perspective, *“[There is a] shortage of ambulances…. This affects referral services. [You] can’t run from one end to another at some points. [So] a mother’s life may not be saved because there’s not enough time”.*

### Poverty: many faces, few choices (socio-economic factors)

The complex cycle of poverty appears more insurmountable at delivery than at ANC. Women who attend ANC often receive incentives such as mosquito nets and “mama kits” (a clean delivery kit that includes plastic sheet, gloves, razor blade, string, clean cloth). In contrast, women who deliver at a health facility do not routinely receive any incentives, but are expected to provide their own items, called “requirements”, such as soap, basin, baby blanket, and other supplies.

Several respondents described the financial and emotional stresses endured by women and families living in poverty. The agony of choice was captured in human terms by one respondent: *“[You] use the money you would have used for your children to go to school [in order] to go to the health facility”.*

As one female health worker shared: *“There are others who really…cannot afford to buy a basin. They feel like when they come with an old basin to the health centre, she will be looked down upon and [people will say], ‘This one is the poorest’ or ‘This one is very ignorant’ …so they normally have that fear of coming with what they have at home".*

Another respondent said, *“People think about going for ANC, but don’t go for health facility delivery because of requirements. If you go without them, you’ll be chased away”.*

The sense of exclusion and “*defeat*” that poverty inflicts on these communities was summed up by a male FGD respondent in seven words: *“Poor people cannot deliver at the hospital”.*

### Socio-cultural and gender barriers: Perceptions and realities

Women understand the need to attend ANC at least once in order to obtain their ANC card as a kind of entry ticket, in case they experience complications at delivery and need health centre assistance. However, women do not perceive delivery at a health facility as equally crucial. There is no alternative to attending ANC clinic for obtaining the ANC card, but women view delivery by traditional birth attendants as an alternative to delivery at a health facility.

Culturally, home delivery is perceived as normal while hospital delivery is considered suitable for the sick or those with complications.

*“The general tendency is for home delivery because it’s more comfortable… and there’s no need for health facility delivery if the woman is not unwell…. The reason to go to health facility is if [she] thinks something may go wrong back home. Women go to ANC to get the card and get registered so they are not blamed if they go to health facility for delivery, in case of complications….” (male policymaker)*

Another female interviewee affirmed this: *“Through ANC service, a woman is told whether the fetus is healthy, in a good position, or doing weakly in the womb. Hence, [she] knows whether [she] has to deliver in hospital or at home.”* Once a woman has given birth at least once, there is a sense that she now knows the dynamics of labour and can henceforth deliver from home.

Both male and female policymakers confirmed this perspective, revealing deeply rooted traditions: *“It’s a general belief that a strong woman can deliver alone. This is also a belief amongst our people here. A strong woman should be able to deliver alone. Or, she should be able to go to the health facility and within two hours get out”* (male policymaker).

As revealed through qualitative data, one Ugandan doctor/policy maker interviewed for this study from outside the study zone had innovated to make his hospital more welcoming. The hospital now encourages women to bring a birth companion to delivery, installs stools so the companion can sit by the bedside of the labouring woman, and organises community “Safe Motherhood Days”, including tours of maternity wards. Based on observations and communications with this doctor, one policymaker indicated that these strategies made a positive difference at a minimal cost and could potentially be replicated elsewhere in Uganda.

### The supply side: human resources management and health systems challenges in implementing a universal SBA policy

Key challenges highlighted by both health workers and policymakers included: addressing staff shortages and working conditions; ensuring staff competencies and training; recruiting, maintaining, and motivating staff, particularly in a region devastated by decades of conflict; staff salaries and housing; ensuring adequate supplies, equipment, and logistics; and providing a functional referral system.

## Discussion

### What health workers say matters: association between advice at ANC and place of delivery

ANC service providers can play a key role in encouraging pregnant women to seek SBA services and increasing the proportion of deliveries at health facility [37]. After controlling for potential confounders, reporting having received ANC advice to deliver at a health facility was significantly associated with reporting having delivered at a health facility. This confirms results from other African settings [38, 39] indicating that the likelihood of delivering in a health facility is significantly higher among women counseled at ANC about possible pregnancy complications than among those not counseled. While this represents a window of opportunity for intervention, just over half of women reported being so advised. While it is possible that women who ended up delivering in a facility recalled being advised to do so to justify their decision, a randomized controlled trial (RCT) including birth planning and advice to deliver in a facility showed the same thing prospectively.

### Quality of care and client-provider interaction as a primary barrier

Of serious concern is women's fear or experience of maltreatment by health workers, which is the barrier to health facility delivery reported most often. This finding echoes others from Uganda [23, 40, 41] and other African countries [42–44, 45]. All these studies underscore the importance of women’s perceptions or expectations of treatment by health workers as a primary factor in deciding whether to deliver at a health facility. Women’s particular vulnerability during childbirth may make them more afraid of such maltreatment during delivery compared to ANC.

There are many ways to help health facilities become more “woman-friendly” and respectful of individual women and their socio-cultural beliefs and traditions. It is not clear whether health workers and policymakers are aware of the extent to which poverty, perceived disrespectful treatment, and lack of respect for traditions prevent women from delivering at health facilities. Such facilities could provide soap, basin, and the other “requirements”, ensure that all women are treated with dignity and respect, encourage women to have a birth companion, provide an area to heat water for bathing, and allow traditional blessings to be performed after birth. Clean birth kits could be provided during delivery not ANC, to make women feel more welcome.

The report on the Ugandan doctor who had made the maternity a more “woman-friendly” environment, implied doing so did not require complex or expensive technology, a major overhaul, or significant costs. Rather, it implied listening to and observing the experience and feelings of women patients, encouraging and supporting staff to embrace change, and monitoring the process and data to observe the impact.

### Access viewed as a major barrier by all groups of respondents

Long distances and lack of transport were barriers cited frequently by all respondents, confirming earlier findings [46–49]. Although the setting was chosen to minimize the role of distance as a barrier, in this post-conflict population, many people left the IDP camps and returned to their home villages where few or no services are available, further decreasing their accessibility. It is important to monitor health facility delivery, since decreasing utilisation could result in deteriorating health outcomes, including birth outcomes. It is crucial to both ensure an adequate supply of skilled birth attendants in this underserved region and for those health workers to counsel all women in post-conflict communities to deliver at health facilities. National and regional policies and programmes should be reviewed to ensure increased, equitable access to health care delivery services in post-conflict communities. This may involve improving roads and transportation options, including ambulances; increasing the number of facilities offering SBA in remote areas; providing financial support or incentives to the poorest; and ensuring cultural sensitivity of services.

### Poverty: from dignity to “defeat”

Poverty can have many faces, its most obvious being the financial constraints. Poverty was flagged as a major and often insurmountable barrier, confirming earlier results from Uganda [50] and elsewhere [43, 46, 49, 51–53].

Our findings echo the call for strategies of financial protection for the poorest [54–56]. Although health services in Uganda, including ANC and delivery services, are officially free – i.e., do not require a fee to be paid –, residual costs may prove insurmountable for the poor or very poor. Not only must women and their families pay the costs of transport to the health facility, they are also expected to purchase certain supplies, i.e., basin, soap, baby clothes, blanket. Similarly, out-of-pocket costs for transportation and “unofficial” provider payments still posed a barrier in Tanzania despite user-fee exemptions [56]. Failure to equip health facilities with essential supplies results in exclusion or discrimination against the poor who cannot provide their own.

User fee exemption policies need to be planned and implemented with awareness toward their impact on clients’ ability to utilise services, as well as on the quality of those services. Subsequent assessments should be carried out to ensure that services remain affordable for the poorest and most vulnerable populations.

Impact evaluations [57, 58] and systematic reviews [59, 60] found strong evidence that conditional cash transfers (CCTs) and user fee removals appear to be effective in increasing access to and utilisation of health services among the poor. Questions remain regarding replicability of CCTs in more deprived contexts, as their success depends on functional, effective health systems that can both provide quality services and reliably handle financial transfers to clients.

In Northern Uganda’s Gulu District, poverty’s second face is scorn and shame. Some women felt defeated by the need to provide their own supplies and did not even attempt to reach a health facility for delivery, feeling it was not worth facing disapproval, humiliation, and rejection by health workers, and the risk of being sent away when they could not afford the expected supplies. Demanding such supplies from poor women exacerbates existing inequities. This approach should be replaced by a “pro-poor” strategy to pro-actively encourage and enable poor women to seek skilled obstetric care. Given that incentives were among the most commonly cited advantages of attending ANC, their role in motivating women to seek delivery at a health facility should not be overlooked.

### What enables women to deliver in health facilities?

Women highlighted the following enabling factors to give birth in a health facility (in order of importance): 1) being fully prepared in advance, including having all expected supplies; 2) receiving incentives; 3) improving the way they are treated by health workers; 4) increasing access to health facilities; 5) strengthening health education and sensitisation among women, families, and communities regarding the importance of health facility delivery; and 6) enhancing male involvement and support during pregnancy, labour, and delivery.

### Birth planning and preparedness, including advice at ANC

The fact that women frequently highlighted planning in advance, being fully prepared and ready with all the needed supplies as a key facilitating factor of health facility delivery merits further attention. One policymaker explained that birth planning was previously introduced in Uganda and appeared promising, but the list of supplies expected by health workers posed a problem, so birth planning seems to have been abandoned.

An RCT in rural Tanzania [61] suggests that health workers making birth plans together with women during facility-based ANC almost doubled the proportion of women giving birth in a health facility (35 % vs. 20 %). It may be worth re-introducing birth plans in Uganda, after improving the availability of supplies at health facilities. If health workers are overworked and cannot establish individual birth plans in the ANC clinic, training village health teams or traditional birth attendants to do birth planning with women at the community level could be an option worth testing.

### Strengthening the supply side: health systems and health workers need greater resources, support

Challenges to the health system and health workers point to the need for the investment of more resources in obstetric care. Where governments increase financing of obstetric care, the proportion of women receiving SBA is likely to increase [56]. The challenges we found reflect those identified by the wider maternal and newborn health community, as highlighted in key documents including *The Lancet* Midwifery series [62], *A Manifesto for Maternal Health Post-2015* [63], and the Global Strategy for Women’s and Children’s Health [64]. All these documents call for substantially increased investment to strengthen health systems in developing countries, including the development of a skilled, motivated health workforce, deployed where most needed and operating within an enabling, well-supervised, and accountable environment.

### Study strengths and limitations

Strengths of this research study include its combination of quantitative and qualitative methods to triangulate research findings and appropriately answer the research questions (1) why the ANC-SBA gap exists, (2) what effect health workers’ advice has on women’s place of delivery, and (3) whether health workers proactively encourage women to deliver in health facilities, and, if so, how. Exploring both supply- and demand-side perspectives, both pre-labour and during labour (as laid out in the conceptual framework) enriches the findings. Restricting the study to a population that had access and where delivery was free, as well as controlling for a number of potential confounders through selection of an MSF-supported site that is fully staffed, equipped, and functional is another key advantage of this study.

The study contributes to the body of knowledge on an important phenomenon within maternal and newborn health, namely, the ANC-SBA gap, in a setting where little substantive maternal health research has been carried out since the start of the civil war 25 years ago.

The significant gap between women’s utilisation of ANC and SBA is not unique to Northern Uganda but is a common phenomenon in many countries, especially in Africa. While our findings may not be generalisable to all settings, they can offer insights and potentially useful recommendations that could be applied and evaluated in other settings.

The study’s main limitation is that data from women interviewees concerning advice received at ANC during previous pregnancy (i.e. the most recent pregnancy, prior to this study) was self-reported retrospectively, not observed or validated, and, thus, possibly subject to recall bias. Place of delivery during last pregnancy was also self-reported and not verified. Such potential bias may have led to an overestimation of the strength of the association between receiving advice to deliver in health facilities and actually doing so. Additionally, the very high proportion of women (98 %) stating their intention to deliver in a health facility may reflect social desirability/courtesy bias and, thus, the figure cannot be taken at face value. Finally, the small sample size of the quantitative study may be considered a potential limitation. Although sample size was calculated to ensure 80 % power to detect an odds ratio of 4 for the primary outcome of delivery at health facility, given advice at ANC to do so, it may not have been adequate to test for statistically significant associations with other covariates or predictors of the primary outcome and exposure.

### Recommendations for future research

The current situation of maternal and newborn health in Northern Uganda calls for further implementation research to address the barriers and facilitators described above. To identify and evaluate specific interventions that may improve the situation, potentially useful studies include:

- An analysis of why health workers do not consistently and universally advise ANC clients to deliver at health facilities; whether they have made the shift from the high-risk profiling approach in ANC to promoting SBA for all; and whether they are aware of the ANC-SBA gap in their own health facility’s service statistics;
- Testing and comparing the effectiveness, cost-effectiveness, acceptability, and sustainability of various strategies for improving quality of care and client-provider interaction, as perceived by women clients, especially the poor and vulnerable.
- A comprehensive review examining all relevant programmes and policies on maternal and newborn health in Northern Uganda, using a “post-conflict” and a “pro-poor” lens to assess: 1) whether the repatriation has worsened the situation and, if so, how this deterioration of accessibility can be prevented, and 2) how policies and practices (e.g., “requirements”) disadvantage the poor;
- Evaluating various “pro-poor” strategies, such as demand-side financing to assess which ones might effectively alleviate the problem of pervasive poverty in the region and increases uptake of SBA;

A pilot study, conducted in selected sub-counties of Gulu District, of a community-based support and follow-up system to be quickly activated when a woman going into labour needs emergency transport. The follow-up system could involve Village Health Team members and traditional birth attendants to help ensure availability of such transport; monitor and promote women’s attendance at ANC and SBA; and track maternal and neonatal outcomes. A systematic review [65] concluded that evidence is promising, albeit limited, that community referral and transport schemes may increase uptake of SBA and EmOC. If such a pilot proved effective, feasible, and acceptable, a larger trial could be designed and conducted.

## Conclusions

Initiatives to improve quality of client-provider interaction and respect for women are essential. Financial barriers (including but not limited to “requirements”) must be abolished and emergency transport for referrals improved. Simultaneously, supply-side barriers must be addressed, notably ensuring a sufficient number of health workers providing skilled obstetric care in health facilities and creating livable conditions and enabling environments for them.

## Abbreviations

ANC: Antenatal Care
CCT: Conditional Cash Transfer
EmOC: Emergency Obstetric Care
FGD: Focus Group Discussion
IDP: Internally Displaced Persons
MSF: Medecins Sans Frontieres/ Medicos Sin Fronteras
OR: Odds Ratio
RCT: Randomized Controlled Trial
SBA: Skilled Birth Attendance
WHO: World Health Organization
